# Medical Scribes in the Emergency Department: The Scribes’ Point of View

**DOI:** 10.31486/toj.18.0176

**Published:** 2019

**Authors:** Robert M. Eley, Brandon R. Allen

**Affiliations:** ^1^ Emergency Department, Princess Alexandra Hospital, Woolloongabba, Queensland, Australia; ^2^ Faculty of Medicine, The University of Queensland, Brisbane, Queensland, Australia; ^3^ Department of Emergency Medicine, University of Florida College of Medicine, Gainesville, FL

**Keywords:** *Emergency service–hospital*, *medical scribes*, *students–medical*, *students–premedical*

## Abstract

**Background:** Studies report the benefit of medical scribes in the emergency department on patient throughput, clinical documentation, patient outcomes, and provider and patient satisfaction. However, studies are silent on the benefits of being a scribe for premedical and medical students.

**Methods:** The senior author interviewed 8 scribes who were applying for medical school and 9 medical students who had been scribes prior to medical school. Discussion was prompted on undergraduate education; scribe recruitment and training; career intentions; experience as a scribe; and the value of being a scribe to themselves, to the doctors with whom they worked, and to the hospital where they were employed.

**Results:** The typical scribe had become a scribe to support his or her chances of entry into medical school. Those already in medical school were not convinced that this experience had actually made a difference in their acceptance. All 17 scribes were emphatic that the role had benefitted them in other ways, specifically, by learning medical terminology, observing communication between doctor and patient, and understanding the practice of medicine in an emergency department. For many scribes, the experience reinforced the desire to become a doctor. The scribes recognized their value in the areas of process and finance. They also recognized that many doctors, particularly those working in academic health centers, derived satisfaction from the training and mentoring that they offered.

**Conclusion:** Scribes perceive the role of a scribe to be highly valuable in terms of their career decision making and future medical education.

## INTRODUCTION

After they are trained in medical terminology, charting methods, physical assessment, clinical investigations and interpretation of findings, elements of professionalism, and bedside etiquette, scribes follow doctors during patient care to record patient data and procedures. Since early reports of their introduction into the emergency room,^1,2^ scribes have become a common feature across the US health system.^3^ Driven in part by the implementation of electronic health records and their increased documentation requirements, the use of scribes is on the increase.^4^ The American College of Medical Scribe Specialists estimates an increase from 20,000 scribes in 2015 to 100,000 by 2020.^4^ Despite the popularity of scribes in the United States, their use is not common outside of the US health system; however, the use of scribes has been described in Canada^5^ and Australia.^6,7^

**Figure 1.**
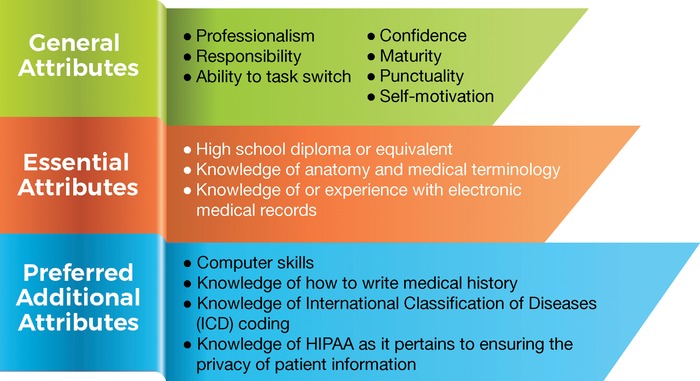
**General requirements for entry into scribe training collated from websites of training institutions.** HIPAA, Health Insurance Portability and Accountability Act.

Training of medical scribes in the United States follows 3 main pathways. The first is training by 1 of the more than 20 commercial companies^4^ contracted to provide scribes to health providers. The second is ad hoc training in an individual hospital or health system to serve the institution's own requirements. The third is training in a formal hospital-based program that has a primary focus to mentor and train premedical (premed) students.^8^ Prerequisites for entry in a scribe training program vary considerably. The minimum requirements range from a high school diploma to an undergraduate degree preferred, as well as a variety of general attributes (Figure 1).

The benefits of scribes in the emergency setting have been described.^9-11^ Benefits have been demonstrated in improvements to patient throughput; documentation; patient outcomes; hospital billing and revenue; resident training; and provider, consultant, and patient satisfaction.^6,12,13^ Although not every study demonstrated that scribes provided a significant benefit in terms of the study's primary outcome (eg, length of stay), no study reported inferior outcomes associated with the use of scribes.

Despite the number of articles describing the value of scribes, articles focused on the value of being a scribe are few. Those that exist are limited to interviews,^14^ opinion pieces,^15^ blogs,^16^ letters,^17^ and recruitment programs.^18^ The most complete work is a 2017 doctoral thesis by Lowry that examined being a scribe from the scribes’ perspective.^19^

This study complements Lowry's work by presenting the findings from interviews with medical (med) students who worked as scribes before entering medical school. However, this study adds new insight from interviews with current scribes who were aspiring to enter medical school.

## METHODS

We conducted 17 interviews with current and past scribes. Eight interviews were conducted with current scribes who had completed their undergraduate degrees in the past 12 months and had intentions of applying to medical school (premed cohort). Seven members of this cohort had been trained to scribe in a formal training program in an academic hospital setting, and 1 was trained through a commercial organization.

The other 9 interviewees were medical students who had been scribes prior to entering medical school (med cohort). All but 1 member of this cohort had been trained by commercial organizations and had been employed as scribes at various locations in the United States. The exception was a medical student from New Zealand who had worked in a private Australian hospital after informal on-the-job training.

The senior author (R.M.E.) conducted each interview in person. The med cohort interviews took place in January 2018 and the premed interviews in July 2018. A narrative semistructured interview method was used that allowed flexibility based upon the direction of the participant's responses. The semistructured interviews started with the collection of information about the individual's undergraduate education and his/her recruitment and training to be a scribe. Once that background was established, a framework of 6 questions elicited responses on the following themes:

- Theme 1: Reasons for becoming a scribe
- Theme 2: Effect of being a scribe on career path
- Theme 3: Value of being a scribe in acceptance to medical school
- Theme 4: Value of being a scribe
- Theme 5: Relationships with and value to the doctors with whom they worked
- Theme 6: Value to the hospital where they were employed

Each interview, which took between 20 and 30 minutes, was recorded and transcribed for subsequent analysis that adopted an interpretive account of the responses, using the methodology of Braun and Clarke.^20^ Through this approach, subthemes within the themed framework were identified. The analysis was conducted by the senior author (R.M.E.) and reviewed by the second author (B.R.A.). Queries were discussed, and reporting was based on agreement.

The study was approved by the Metro South Health Human Research and Ethics Committee (HREC/16/QPAH/284), and each of the participants consented to interview.

## RESULTS

### Background

Scribes’ ages ranged from 22 to 28 years; 9 were female. All had worked as scribes for 6 months to 2 years either part time or full time. The entire premed cohort intended to continue in the role until they were accepted into medical school. Prior to becoming scribes, they had either no hospital experience or limited hospital experience through volunteering and shadowing doctors.

Timing for becoming a scribe varied between junior year and postgraduation. Knowledge about the position came from a variety of sources, including scribe company advertising on campus; information from a friend, relative, or classmate; and seeing scribes in action while volunteering at a hospital.

### Theme 1: Reasons for Becoming a Scribe

**Figure 2.**
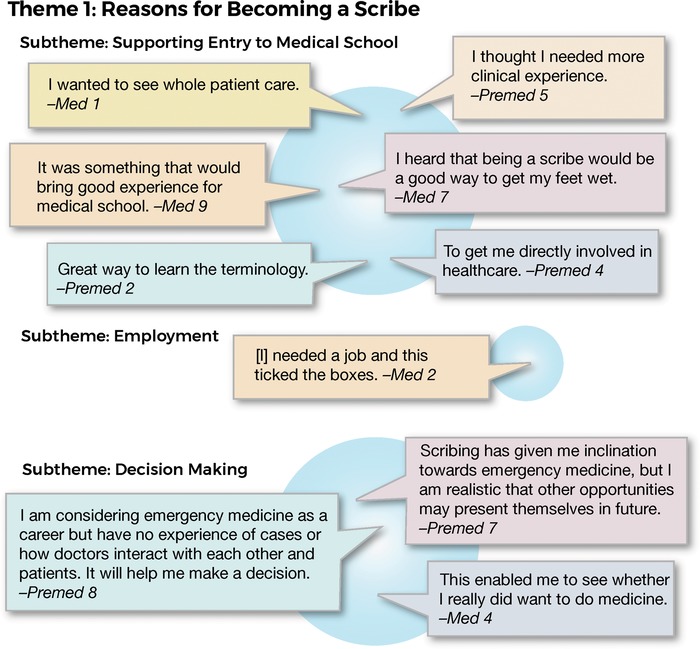
**Interview responses related to theme 1: reasons for becoming a scribe.**

The major subtheme that emerged in response to the question about the students’ reasons for becoming a scribe was supporting entry to medical school. Becoming a scribe was a strategic decision to achieve that goal. Additional subthemes were employment and decision making as to whether or not to pursue medicine or to plan a career pathway. Several students were thinking several years ahead as to their future specialty. Specific responses related to each subtheme are shown in Figure 2.

### Theme 2: Effect of Being a Scribe on Career Path

**Figure 3.**
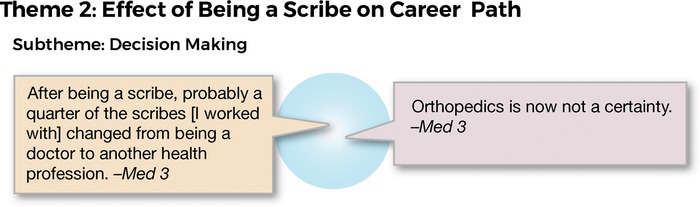
**Interview responses related to theme 2: effect of being a scribe on career path.**

Decision making was also a subtheme that emerged in response to the line of enquiry about the effect of being a scribe on career path (Figure 3). None of the participants indicated that their desire to become a doctor had changed as a result of being a scribe; however, one of the med cohort interviewees stated, “After being a scribe, probably a quarter of the scribes [I worked with] changed from being a doctor to another health profession.” The decision to leave medicine was not corroborated by any of the others who were interviewed. In fact, 3 interviewees stated that they had changed their career pathway from physician assistant to doctor. Several of the participants revealed that their interests changed as a result of the scribe experience. For example, a medical student stated, “Orthopedics is now not a certainty.”

### Theme 3: Value of Being a Scribe in Acceptance to Medical School

**Figure 4.**
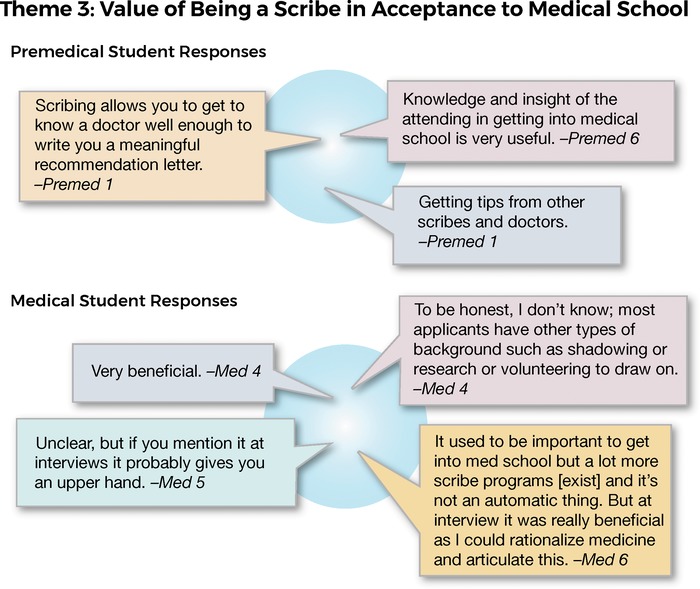
**Interview responses related to theme 3: value of being a scribe in acceptance to medical school.**

When asked specifically about the value of being a scribe in supporting entry to medical school, the premed cohort believed that being a scribe would be beneficial in their preparation and application process. In particular, they noted the support from the doctors who assisted in preparing them for the Medical College Admission Test. While all members of the med cohort had become scribes to assist in medical school entry, in retrospect, they were not convinced of its value. Representative comments from both cohorts are shown in Figure 4.

### Theme 4: Value of Being a Scribe

**Figure 5.**
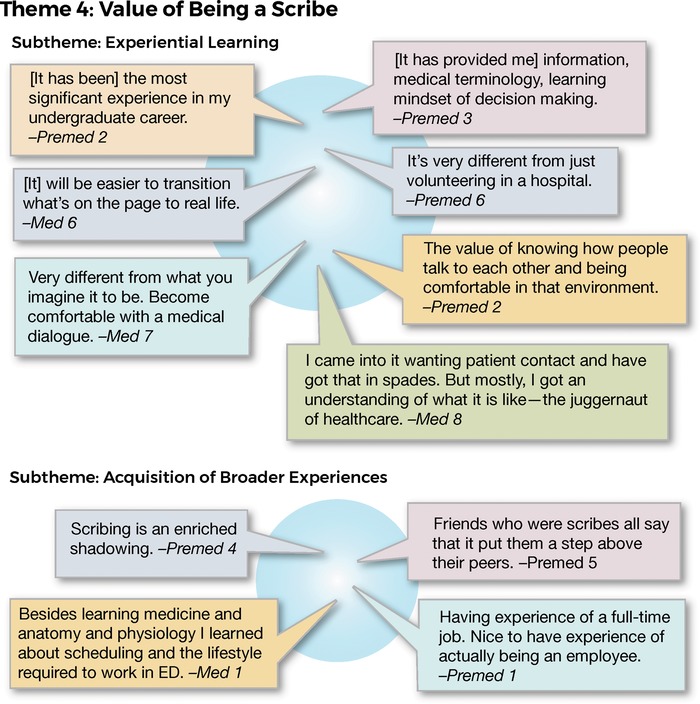
**Interview responses related to theme 4: value of being a scribe.**

Regardless of the value of the scribe experience in terms of medical school entry, all 17 interviewees were emphatic that being a scribe had been/was of great benefit to them with outcomes exceeding expectations. The major subtheme that emerged was experiential learning. Acquisition of broader experiences was a secondary subtheme in the responses to the question about the value of being a scribe. Representative responses are shown in Figure 5.

**Figure 6.**
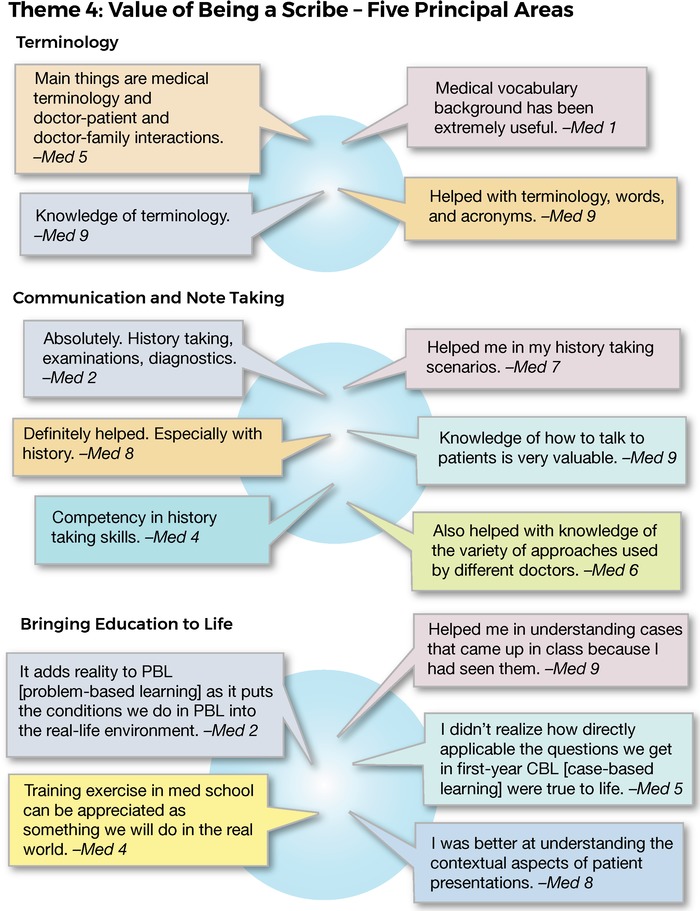

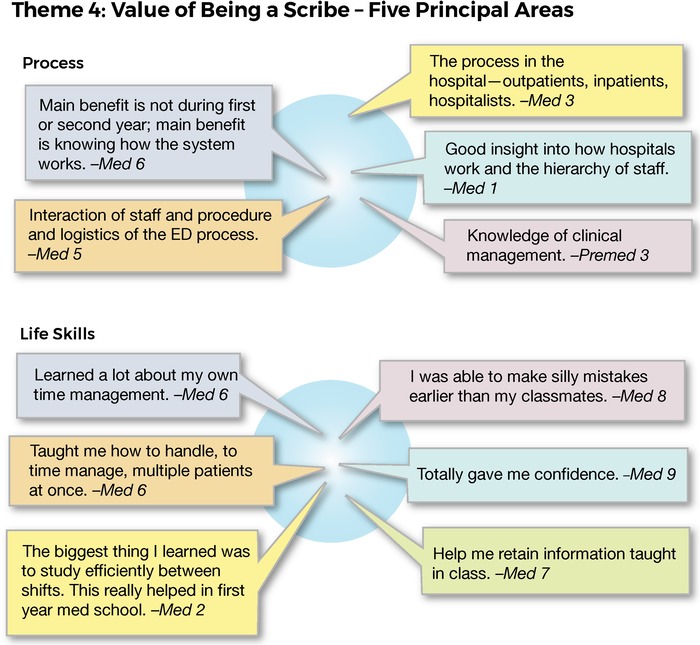
**Interview responses identifying 5 principal values related to theme 4: value of being a scribe.**

These views were reinforced by statements from the med cohort who extolled the value they had derived from being a scribe in 5 principal areas: terminology, communication and note taking, bringing education to life, process, and life skills. Specific responses are shown in Figure 6.

### Theme 5: Relationships With and Value to the Doctors With Whom They Worked

**Figure 7.**
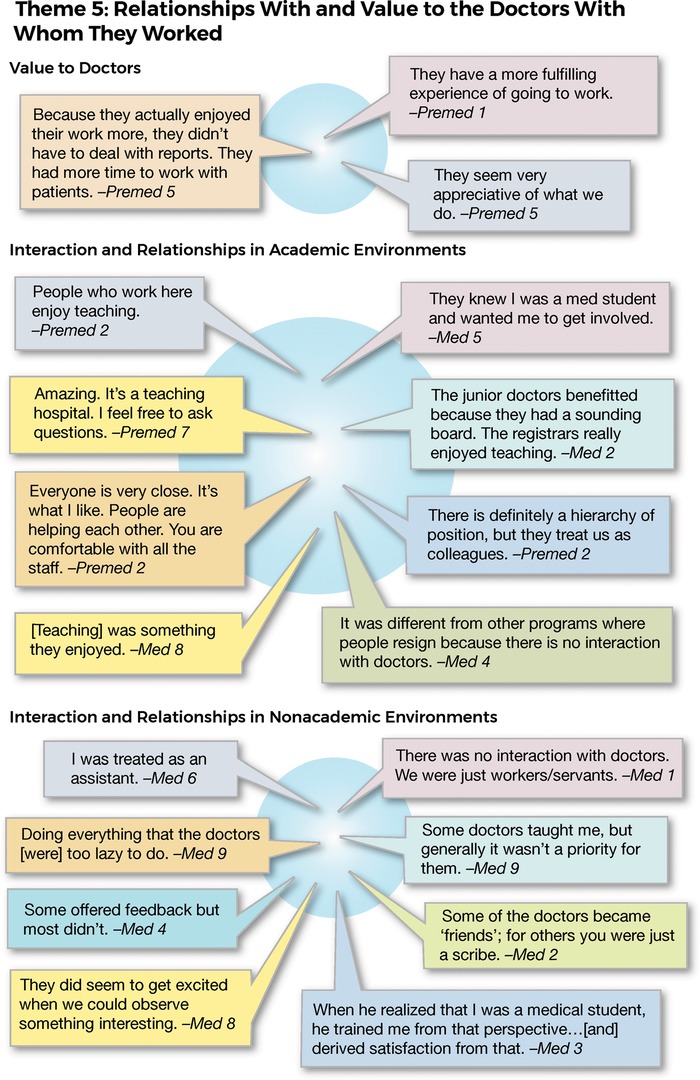
**Interview responses related to theme 5: relationships with and value to the doctors with whom they worked.**

All those interviewed recognized the value to doctors of having scribes. However, some differences of opinion emerged between scribes who had worked or were working in a university health system compared to those working outside of the academic environment. The former group recognized that the doctors with whom they worked were familiar with roles of teaching and mentoring in an academic environment as indicated by the responses in Figure 7. Scribes who worked in academic environments consistently reported close interaction and strong relationships with medical staff. In contrast, some of those employed in nonacademic departments noted no real interaction between themselves and the medical staff in the role of employer/employee. Furthermore, support in terms of teaching and mentorship was not always forthcoming. However, these views were not universal; several interviewees noted a “teacher, mentor relationship,” and one scribe who worked in a nonacademic environment reported close interaction with a doctor: “When he realized that I was a medical student, he trained me from that perspective.” The student noted that the doctor “…derived satisfaction from that.”

### Theme 6: Value to the Hospital Where They Were Employed

**Figure 8.**
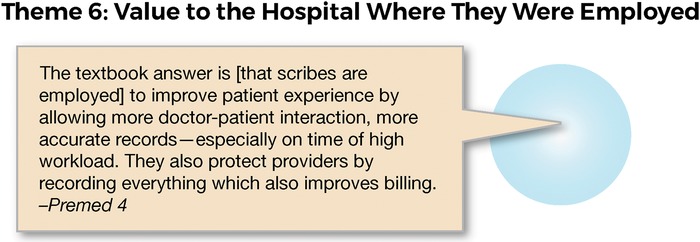
**Interview response related to theme 6: value to the hospital where they were employed.**

The scribes were all aware that their position benefitted the emergency department and the hospital. These benefits were in the areas of system processes (reduction in wait times, improved patient flow) and improved patient care (quality of doctor-patient interactions, potentially more accurate reporting). These benefits are summarized in the representative response shown in Figure 8.

### Negatives of Being a Scribe

The interviewees reported few negatives associated with being a scribe. The subtheme of time management, especially related to long shifts, emerged in the responses of a couple of interviewees. A member of the premed cohort mentioned the “challenge of [a] long shift especially when it was a part-time job.” But this was also seen as an opportunity. One of the med cohort interviewees noted, “It taught me a great deal of how to manage time.”

Consistently, pay was described as being “miserable” but was never of great concern.

## DISCUSSION

The objective of this study was to evaluate the scribes’ perceived benefit of being a trained scribe. Both cohorts considered that their role as scribe was valuable to them. Being a scribe reinforced their career plan, provided knowledge and context for medical school, and assisted in the acquisition of life skills. Specifically, value was acquired in knowledge of medical terminology, doctor-patient and doctor-family interactions, hospital processes, history taking, and understanding the contextual aspects of patient presentations. Understanding the real-life context during their medical education was seen as major boon. The results support the doctoral work of Lowry^19^ and the commentaries by others.^14-18^

We found great similarity between the med and premed cohort responses except in the perceived value of being a scribe to medical school entry. The differences could largely be attributed to hindsight. The premed cohort strongly believed that being a scribe would aid them in getting into medical school. This view is promoted by the chief executive officer of ScribeAmerica who was quoted saying, “A background in medical scribing is quickly becoming the standard for pre-medical experience and is suggested by medical school acceptance committees across the country.”^21^ However, the scribes who were already in medical school were unconvinced that being a scribe was a major advantage. They recognized that many, if not all, students with aspirations to go to medical school engage with the health system in one capacity or another, and being a scribe was not seen to be any more advantageous in this regard than volunteering, shadowing, or undertaking research.

Differences were also apparent between scribes who had been trained and employed in the commercial context and those who trained in a program designed to support premedical students in their career pathway and decision-making process. The mentoring and teaching consistently provided to the latter group were inconsistently available to the former group. Although of interest and noted, comparing training programs or employment was not an objective of this study. It is therefore sufficient to note that although the 2 paths are different in philosophy, the final products (ie, trained scribes) did not differ in the majority of their views or the value they ascribed to that role in their career development.

The scribes understood their value to their employers in assuring complete and accurate documentation and thus improving hospital billing and revenue and potentially improving patient outcomes. They also recognized the increased quality of doctor-patient interaction. These values are consistent with those reported in the literature.^9-11^

To our knowledge, this is the first peer-reviewed paper reporting on this topic. Our findings reinforce the doctoral work of Lowry^19^ but add the views of premed students. Limitations of the study are the relatively small number of participants and that the members of the premed cohort were all working in the same environment. However, the similarity of the responses between the premed and med cohorts (each member of the med cohort had worked in different environments) suggests that the limitations were not major.

## CONCLUSION

The value of the scribe program is far greater than the system benefits afforded to health service providers. The perceived value to our future doctors is high. Whether the experience results in better performance during medical school in areas such as clinical reasoning or eventually manifests beyond medical school are questions that would be interesting to pursue.

## Notes

This article meets the Accreditation Council for Graduate Medical Education and the American Board of Medical Specialties Maintenance of Certification competencies for Patient Care, Medical Knowledge, Interpersonal and Communication Skills, and Professionalism.
